# Diphtheria toxin induced but not CSF1R inhibitor mediated microglia ablation model leads to the loss of CSF/ventricular spaces in vivo that is independent of cytokine upregulation

**DOI:** 10.1186/s12974-021-02367-w

**Published:** 2022-01-04

**Authors:** Alicia Bedolla, Aleksandr Taranov, Fucheng Luo, Jiapeng Wang, Flavia Turcato, Elizabeth M. Fugate, Nigel H. Greig, Diana M. Lindquist, Steven A. Crone, June Goto, Yu Luo

**Affiliations:** 1 Department of Molecular Genetics and Biochemistry, College of Medicine, University of Cincinnati, Cincinnati, OH USA; 2 Imaging Research Center, Cincinnati Children’s Hospital Medical Center, Department of Radiology, University of Cincinnati, Cincinnati, USA; 3 Drug Design & Development Section, Translational Gerontology Branch, Intramural Research Program, National Institute On Aging, National Institutes of Health, Baltimore, USA; 4 Division of Neurosurgery, Cincinnati Children’s Hospital Medical Center, 3333 Burnet Avenue, Cincinnati, OH 45229 USA; 5 Division of Developmental Biology, Cincinnati Children’s Hospital Medical Center, 3333 Burnet Avenue, Cincinnati, OH 45229 USA; 6 Department of Neurosurgery, College of Medicine, University of Cincinnati, 231 Albert Sabin Way, Cincinnati, OH 45267 USA

**Keywords:** Microglia, Genetic diphtheria toxin receptor ablation model, Genetic Cx3cr1CreER model, Edema, Neuroinflammation, Choroid plexus

## Abstract

**Background:**

Two recently developed novel rodent models have been reported to ablate microglia, either by genetically targeting microglia (via Cx3cr1-creER: iDTR + Dtx) or through pharmacologically targeting the CSF1R receptor with its inhibitor (PLX5622). Both models have been widely used in recent years to define essential functions of microglia and have led to high impact studies that have moved the field forward.

**Methods:**

Using either Cx3cr1-iDTR mice in combination with Dtx or via the PLX5622 diet to pharmacologically ablate microglia, we compared the two models via MRI and histology to study the general anatomy of the brain and the CSF/ventricular systems. Additionally, we analyzed the cytokine profile in both microglia ablation models.

**Results:**

We discovered that the genetic ablation (Cx3cr1-iDTR + Dtx), but not the pharmacological microglia ablation (PLX5622), displays a surprisingly rapid pathological condition in the brain represented by loss of CSF/ventricles without brain parenchymal swelling. This phenotype was observed both in MRI and histological analysis. To our surprise, we discovered that the iDTR allele alone leads to the loss of CSF/ventricles phenotype following diphtheria toxin (Dtx) treatment independent of cre expression. To examine the underlying mechanism for the loss of CSF in the Cx3cr1-iDTR ablation and iDTR models, we additionally investigated the cytokine profile in the Cx3cr1-iDTR + Dtx, iDTR + Dtx and the PLX models. We found increases of multiple cytokines in the Cx3cr1-iDTR + Dtx but not in the pharmacological ablation model nor the iDTR + Dtx mouse brains at the time of CSF loss (3 days after the first Dtx injection). This result suggests that the upregulation of cytokines is not the cause of the loss of CSF, which is supported by our data indicating that brain parenchyma swelling, or edema are not observed in the Cx3cr1-iDTR + Dtx microglia ablation model. Additionally, pharmacological inhibition of the KC/CXCR2 pathway (the most upregulated cytokine in the Cx3cr1-iDTR + Dtx model) did not resolve the CSF/ventricular loss phenotype in the genetic microglia ablation model. Instead, both the Cx3cr1-iDTR + Dtx ablation and iDTR + Dtx models showed increased activated IBA1 + cells in the choroid plexus (CP), suggesting that CP-related pathology might be the contributing factor for the observed CSF/ventricular shrinkage phenotype.

**Conclusions:**

Our data, for the first time, reveal a robust and global CSF/ventricular space shrinkage pathology in the Cx3cr1-iDTR genetic ablation model caused by iDTR allele, but not in the PLX5622 ablation model, and suggest that this pathology is not due to brain edema formation but to CP related pathology. Given the wide utilization of the iDTR allele and the Cx3cr1-iDTR model, it is crucial to fully characterize this pathology to understand the underlying causal mechanisms. Specifically, caution is needed when utilizing this model to interpret subtle neurologic functional changes that are thought to be mediated by microglia but could, instead, be due to CSF/ventricular loss in the genetic ablation model.

## Background and significance

Microglia provide the innate component of the brain’s immune system, and their role in maintaining homeostasis of neuronal function under physiological conditions, and mixed dual effects under pathological conditions have become areas of substantial recent interest. During homeostasis throughout both development and adulthood, microglia have been shown to assist in achieving synaptic plasticity by fulfilling the vital role of synaptic pruning [1, 2]. During developmental stages microglia have been shown to exhibit an “activated” state that allows for an ameboid morphology, distinct gene expression, and the release of growth factors necessary for normal brain development [3–5]. This phenotype was shown to promote neuronal and oligodendrocyte differentiation [6]. In adult brain, microglia are known to maintain appropriate physiological conditions through phagocytosis of debris, myelin, and synapses [7, 8]. Additionally, microglia use their purinergic receptors (P2RY12) to find free ATP as a signal to more active cells and moderate synaptic remodeling or maturation [9]. These combined functions are essential for network and neuronal maintenance in the adult brain. While these roles are essential, microglia can also become activated when homeostasis is challenged by injury or disease. Specifically, microglia can adjust to a spectrum of activated phenotypes ranging from a more pro-inflammatory phenotype, which often results in inflammatory damage, to an anti-inflammatory phenotype, offering protection and promoting tissue remodeling and repair [10, 11]. Microglia have been shown to promote disease progression in Alzheimer’s disease, by selective depletion showing better disease outcomes in mice [12, 13]. On the other hand, microglia have also been shown to help regulate calcium levels following stroke injury, resulting in neuronal protection [14]. To understand how microglia affect the function and survival of surrounding neurons under both physiological and pathological conditions, two novel models for ablating microglia in vivo have recently been developed. These either genetically induce microglial cell death [15–17] or pharmacologically target their essential signaling for survival, CSF1 receptor (CSF1R) with its kinase inhibitors, PLX3397 or PLX5562 compounds [12–14, 18–20]. The genetic ablation model utilizes tamoxifen inducible Cre recombinase to genetically (Cx3cr1-CreER) and selectively ablate microglia by activating the expression of the diphtheria toxin receptor (DTR) only in microglia upon administration of tamoxifen, allowing for ablation after the later administration of diphtheria toxin (Dtx). The pharmacological ablation model utilizes a pharmacological inhibitor of the CSF1 receptor (CSF1R), which is critical for microglia function and survival and has minimal effect on peripheral myeloid cells [18, 21]. Both models recently have been widely used to characterize essential functions of microglia across key aspects of neurological function and have led to substantial high impact studies in the field [13–20, 22–25]. Both models effectively ablate microglia specifically; however, each model possesses different features that might favor certain experimental designs over others. The genetic microglia ablation model is rapid (> 80–90% ablation occurs within 2–3 days of Dtx administration) and enables a more precise window of ablation to support time-dependent studies [15, 16]. Contingent on the treatment paradigm (Dtx injection immediately after tamoxifen administration or Dtx injection after 4–6 weeks of tamoxifen administration), this system allows for targeting of either CNS microglia or both CNS microglia and peripheral macrophages [6, 16]. In contrast, the pharmacological ablation model acts by inhibiting the CSF1R (PLX3397 or 5622 compounds) that may take longer to ablate microglia (the first generation compound PLX3397 takes about 3 weeks [18] whereas the specific CSF1R inhibitor- PLX5662 can effectively ablate microglia within 3–7 days [25, 26]). In both ablation models, a repopulation of microglia ensues within 7 days once Dtx or PLX compound is withdrawn [15, 18]. Given the different features of the two models, both have been widely used in many recent studies depending on study design requirements [13–20, 22–25].

Although the microglia ablation models have been demonstrated to be valuable tools in investigating microglia function both under physiological and pathological conditions, it is also important to carefully characterize them to avoid any confounding factors that might affect the interpretation of the data obtained from these models. Here, we discovered and report that the genetic ablation of microglia in Cx3cr1-iDTR model causes a robust unpredicted pathological condition in the brain, characterized by a significant loss of CSF and ventricular spaces. In this study, we aimed to characterize this unique phenotype and to identify the mechanism responsible for the phenotype. Additionally, we examined whether the pharmacological model may, likewise, induce this pathology. We furthermore investigated the cytokine production profile and the reactive astrocyte responses in the two microglia ablation models. Our data suggest that the DTR-mediated genetic microglial ablation model leads to this CSF/ventricular loss pathology that is independent of multiple cytokine upregulation and reactive astrocytes, whereas all these features are absent in the PLX5622-mediated pharmacological microglia ablation model. Lastly, we explored whether the loss of CSF/ventricular space is due to potential brain parenchyma swelling and the contribution of KC/Gro cytokine to this pathology in the genetic ablation model, as we find KC/Gro, contribute to brain edema formation in ischemic stroke, was the most upregulated in the brain of genetic microglia ablation model. Our data indicated that brain parenchyma swelling nor the KC/Gro pathway augmentation is responsible for CSF/ventricular space loss in the genetic microglia ablation model. Instead, to our surprise, we discovered that the ROSA26-iDTR (iDTR) allele is solely responsible for the CSF/ventricular space loss after the Dtx treatment as Dtx-treated iDTR + mice in the absence of any cre expression exhibit the same ventricular phenotype, yet did not show cytokine upregulation or reactive astrocytes. Instead, the choroid plexus (CP), a major source of CSF production, showed increased activated IBA1 + cells in both the iDTR and Cx3cr1-iDTR models after Dtx treatment, suggesting the CP pathology contributes to the observed CSF/ventricular loss.

## Materials and methods

### Animals

All animal experiments were conducted under National Institutes of Health (NIH) Guidelines using the NIH handbook *Animals in Research* and were approved by the Institutional Animal Care and Use Committee (University of Cincinnati). The mice were housed in the animal facility of University of Cincinnati on a 14-h light/ 10-h dark diurnal cycle. Food was provided ad libitum. To genetically ablate microglia, we bred Cx3cr1CreER [27, 28] mice (JAX stock number: 021160) with the ROSA26iDTR [16, 29] (JAX stock number: 007900). Cx3cr1CreER (wt/+): ROSA26iDTR (wt/ +) heterozygous mice (termed Cx3cr1-iDTR mice in this study) are used to achieve microglia ablation following Tamoxifen (TAM) and diphtheria toxin (Dtx) treatment, and Cx3cr1CreER (wt/+): ROSA26iDTR (wt/wt) mice (termed Cx3cr1-Cre mice in this study) or ROSA26iDTR only mice (termed iDTR mice in this study) are used as separate control groups that were subjected to the same TAM + Dtx or Dtx only (for iDTR mice) treatment. Mice were genotyped for the Cx3cr1CreER allele using the PCR (primer sequence: AGG ATG TTG ACT TCC GAG TTG; AAG ACT CAC GTG GAC CTG CT; CGG TTA TTC AAC TTG CAC CA) and ROSA26iDTR (primer sequence: GGA GCG GGA GAA ATG GAT ATG; AAA GTC GCT CTG AGT TGT TAT; GCG AAG AGT TTG TCC TCA ACC).

### Ablation of microglia with Tamoxifen and Diphtheria toxin administration

Female and male mice were given tamoxifen (TAM) dissolved in 10% EtOH/90% sunflower oil by gavage feeding at a dose of 180 mg/kg daily for 5 consecutive days. This dosing regimen was previously demonstrated to provide maximal recombination with minimal mortality [30–33]. For induction of adult microglia ablation, mice were treated with TAM at the age of 6–8 weeks to turn on the expression of Dtx receptor in Cx3cr1 + cells with a waiting period of 4–6 weeks to allow replenishment of DTR-negative peripheral macrophages. According to previous studies, this treatment timeline allowed the labeling of microglia with DTR specifically [15, 16]. At 4–6 weeks after TAM treatment, control and Cx3cr1-iDTR mice were treated with diphtheria toxin (i.p. 20 ng/g/day) for 3 consecutive days to ablate microglia. For ROSA26iDTR only mice, at the age of 10–12 weeks, they were treated with same dosage of diphtheria toxin without TAM treatment (iDTR mice with or without TAM showed a similar phenotype). Animals were then subjected to subsequent analysis, as described below.

### Ablation of microglia using PLX5622

Female and male mice had their diets completely replaced with either PLX5622 diet (AIN-76A Rodent Diet With 1,200 PPM PLX5622, formulated by Research Diets with PLX5622 provided by Plexxikon) or the control diet (AIN-76A Rodent Diet, Research Diets, NJ) for 7 days. Animals had ad libitum access to the diet and water for the entire course of the study.

### MRI and brain and ventricular size measurement

Control and microglia ablated mice were subjected to an imaging session at 1 day before Dtx injection and after the last Dtx injection or before and after 7 days of either PLX5622 or control diet by staff that were blinded to the genotypes/treatment groups. MRI studies were performed on a vertical wide bore 9.4 T Bruker Avance III HD scanner with a 36 mm proton volume coil. T_2_-weighted anatomical coronal images of the brain were acquired with a fat suppressed 2D rapid acquisition with relaxation enhancement (RARE) sequence using the following parameters: TR 4 s, effective TE 71.5 ms, echo spacing 6.5 ms, 9 slices, slice thickness/gap 0.75/0.3 mm, RARE factor 20, receiver bandwidth 67 kHz, averages 4, reconstruction matrix 192 × 192, acquisition matrix 192 × 180, field-of-view (FOV) 28.4 mm × 28.4 mm, and total scan time 2:24 min. Respiratory-triggered 2D diffusion tensor image (DTI) data were acquired using a diffusion-weighted spin-echo echo planar imaging method with a TR of 7750 ms, TE 24 ms, 8 segments, 2 averages, receiver bandwidth 200 kHz, acquisition matrix 114 × 112, reconstruction matrix 114 × 114, FOV 25.6 mm × 25.6 mm. 5 slices, slice thickness/gap 0.5/0.3 mm, 6 diffusion directions, b-value 1000 s/mm^2^ with a single B0 image, scan time approximately 20 min. Finally, a non-triggered 3D RARE sequence was acquired with TR 1800 ms, effective TE 84 ms, echo spacing 12 ms, RARE factor 16, reconstruction matrix 160 × 92 × 92, acquisition matrix 160 × 80 × 92, FOV 32 mm × 18.4 mm × 18.4 mm, receiver bandwidth 66 kHz, 1 average, scan time 13:48.

Ventricular volumes were quantified by drawing and measuring ventricular space for each animal from the individual T2-weighted coronal images (sections ranging from Bregma AP: 1.5 mm to AP: − 4 mm) using ImageJ. Diffusion data were imported into DTI Studio [34]. Raw diffusion images were inspected and those with motion were discarded before calculating the diffusion parametric maps, which were saved and imported into ImageJ for ROI-based analysis. ROIs were drawn in the striatum, cortex, and corpus callosum. Apparent diffusion coefficients (ADCs) for the three regions were extracted from the trace image exported from DTI Studio. Directional metrics for the corpus callosum (fractional anisotropy (FA), radial diffusivity (RD), and axial diffusivity (AD)) were extracted from the corresponding parametric maps. Total brain volume was determined from the 3D acquisition using the FMRIB Software Library [35]. The raw data were imported into ImageJ, cropped to exclude the majority of non-brain tissue, and rescaled by a factor of 10. The cropped and scaled data were then used as inputs to the FSL brain extraction tool (BET). The output from BET was selected to be two files corresponding to brain tissue and CSF. Brain tissue volume in pixels was extracted using the fslstats command in FSL and converted to volume by multiplying by the resolution in mm of the acquisition.

### Histological quantification of brain ventricles

Mice were perfused transcardially with cold phosphate buffer followed by a solution of 4% paraformaldehyde (PFA, pH 7.2) in 0.1 M phosphate buffer (pH 7.2). Brains were removed from the skull, post-fixed in 4% PFA overnight at 4 °C, and sequentially transferred to 20 and 30% sucrose in 0.1 M phosphate buffer (pH 7.2) solutions overnight. Brains were frozen on dry ice and sectioned on a cryostat to obtain coronal sections of 30 μm in thickness. Brain sections were then mounted and cover-slipped. The brain sections were scanned using a PathScan microslide scanner to obtain images of brain sections from + 0.5 mm to − 2.0 mm in reference to Bregma, according to a coronal atlas of the mouse brain (Franklin and Paxinos, (Franklin KBJ 1997)). The brain ventricles were quantified in these histological sections for each animal in ImageJ, similar to the MRI quantification. Group and treatment information was blinded to the image analyzer.

### Immunohistochemistry

The brain sections were harvested as described above. The sections were then incubated with blocking buffer for one hour. The primary antibodies were prepared in the blocking buffer and the sections were incubated in the solution overnight: Rabbit anti-IBA1 (1:1000; Wako) and mouse anti-GFAP (1:1000, Sigma Aldrich). The Mouse anti-NeuN (1:500, Millipore Sigma) primary antibody was incubated for 2 overnights (48-h). After incubation with primary antibody solution for 18–48 h, the sections were washed and incubated for 4 h at room temperature in diluted secondary antibody prepared with blocking solution (secondary antibody conjugated with Alexa 488 or Alexa 555, 1:1000; Life Technologies, Carlsbad, CA, USA). The slides were then washed with phosphate buffer (pH 7.2) and cover-slipped. Images were acquired using a Zeiss LSM 710 Live DUO confocal microscope. Omission of primary or secondary antibodies resulted in no staining and served as negative controls. To quantify total number of IBA1 + microglia, cortical images at 200 × magnification were taken for each animal. Three images per animal from 3 sections containing motor cortex were taken from all animals for quantification, as measured using NIS-Element software on the same three brain sections for each animal (results were consistent in all areas of the brain). These quantification results were averaged for each animal from the measured sections, and used as a single data point for statistical analysis. Group and treatment information were blinded to the image analyzer. Similar to parenchymal IBA1 quantification, for IBA1 counts in the choroid plexus, images were taken at 20 × objective (200 × magnification). Three images per animal were taken of intact choroid plexus in the lateral ventricles, and measured using ImageJ software. The quantification results were averaged for each animal and the average was used for statistical analysis. Group and treatment were blinded to the image analyzer.

### Stereological quantification of NeuN staining

Unbiased stereological counts of neurons labeled using NeuN DAB immunohistochemistry was undertaken as previously described [36]. Briefly, contours of layers I, II/III, IV, V, VI were drawn at 5 × objective in the somatosensory and motor cortex, based on cell density and soma size. Optical fractionator sampling was performed on a Leica DM5000B microscope (Leica Microsystems, Bannockburn, IL) with a motorized stage at 40 × objective. Section thickness was assessed at each sampling site with a 2.5 µm guard zone at the top and bottom of the section. Grid size was (X) 220 µm, (Y) 166 µm and the counting frame was (X) 50 µm, (y) 50 µm, with a depth of 25 µm. Gunderson coefficients of error for *m* = 1 was less than 0.10 for each section. The Stereo Investigator software performed stereological estimates with equal parameters for each layer in Cx3Cr1-Cre, Cx3cr1-iDTR, and iDTR mice (*n* = 3 for each genotype).

### Brain water content analysis

Brains were removed from the skull and 2 mm-thick sections were extracted from the left side of each brain (Bregma AP: 1 mm to -1 mm). The brain samples were weighed and then baked at 65 ^°^C until their weight was constant [37]. The weights were then used to calculate percentages of water weight based on the weight difference after the tissue was fully dried (% of water content = (wet weight−dry weight)/wet weight × 100).

### Gene expression and qRT-PCR

To examine gene expression, brain tissues were harvested from control or Cx3cr1-iDTR mice at 1 day after the last day of Dtx injection, or from mice that were fed with either PLX5622 or control diet for 7 days using the methods described in our previous studies [33, 38, 39] for quantitative RT-PCR. Mice were perfused with phosphate buffer (pH 7.2) before being euthanized, and the brains were immediately removed and chilled on ice. The cortex or striatum was dissected and total RNA was extracted following the instructions from the manufacturer (RNAqueous RNA extraction kit, Thermo Fisher), as described previously [40]. Total RNA (1 μg) was treated with RQ-1 RNase-free DNase I and reverse transcribed into cDNA using random hexamers by Superscript III reverse transcriptase (Life Sciences). cDNA levels for HPRT1 (hypoxanthine phosphoribosyl transferase 1), Hmbs (hydroxymethylbilane synthase) and various target genes were determined, using specific universal probe Library primer probe sets (Roche), by quantitative RT-PCR using a Roche Light Cycler II 480. Relative expression levels were calculated using the delta Ct method compared to Hmbs as a reference gene. Primers and 6-carboxyfluorescein (FAM) labeled probes used in the quantitative RT-PCR for each gene are listed in Table 1. Cortex data are presented in Figures, and striatum data provided similar results.

**Table 1:** Primer/Probe used for qRT-PCR

Primer/probe set		
*Hmbs*	Forward Primer	5'-TCC CTG AAG GAT GTG CCT AC-3'
	Reverse Primer	5'-ACA AGG GTT TTC CCG TTT G-3'
	Probe	Universal Probe Library: Probe 79—Roche
*Hprt1*	Forward Primer	5'-TGA TAG ATC CAT TCC TAT GAC TGT AGA-3'
	Reverse Primer	5'- AAG ACA TTC TTT CCA GTT AAA GTT GAG-3'
	Probe	Universal Probe Library: Probe 22—Roche
*Iba1*	Forward Primer	5'-GGATTTGCAGGGAGGAAAA -3'
	Reverse Primer	5'- TGGGATCATCGAGGAATTG-3'
	Probe	Universal Probe Library: Probe 3—Roche
*Gfap*	Forward Primer	5'- TGGAGGAGGAGATCCAGTTC-3'
	Reverse Primer	5'- AGCTGCTCCCGGAGTTCT-3'
	Probe	Universal Probe Library: Probe 69—Roche
*TNFα*	Forward Primer	5'- CTGTAGCCCACGTCGTAGC-3'
	Reverse Primer	5'- TTGAGATCCATGCCGTTG-3'
	Probe	Universal Probe Library: Probe 25—Roche
*IL-1β*	Forward Primer	5'- AGTTGACGGACCCCAAAAG-3'
	Reverse Primer	5'- AGCTGGATGCTCTCATCAGG-3'
	Probe	Universal Probe Library: Probe 38—Roche
*IL6*	Forward Primer	5'- GCTACCAAACTGGATATAATCAGGA-3'
	Reverse Primer	5'- CCAGGTAGCTATGGTACTCCAGAA-3'
	Probe	Universal Probe Library: Probe 6—Roche
*KC/GRO*	Forward Primer	5'- ACTCCAACACAGCACCATGA-3'
	Reverse Primer	5'- TGGTCTGCAGGCACTGAC-3'
	Probe	Universal Probe Library: Probe 49—Roche

### Cytokine level measurement

Cortex and striatum tissues were dissected from control and Cx3cr1-iDTR mice at 1 day after the last Dtx injection or after 7 days of either PLX5622 or control diet, and were prepared in MSD Tris lysis buffer (#R60TX-3) according to the manufacture’s instruction. Cytokine levels were measured using the V-PLEX Mouse Cytokine Plex panel, which includes IFN-γ, IL-10, IL-12p70, IL-1β, IL-2, IL-4, IL-5, IL-6, KC/GRO, TNF-α (Cat#: K15048D, Meso Scale Discovery). Protein concentration was measured by the BCA (bicinchoninic acid assay) method for each sample. Total cytokine levels were normalized to the protein concentration in each sample (each with 3 technical replicates).

### Repertaxin treatment in the genetic microglia ablation model

To investigate whether Repertaxin treatment (a KC/Gro pathway inhibitor) can reverse the CSF/ventricular space shrinkage phenotype in the genetic microglia ablation model, mice were treated with vehicle or Repertaxin at 6 h after each Dtx injection at a dose of 15 mg/kg, based on previous research characterizing Repertaxin’s dose-dependent effects in vivo [41–43]. Our pilot study using this dosage in an ischemic stroke model demonstrated efficacy in reducing edema (Fig. 6A–D). At 24 h after the last Dtx injection, T2-weighted images were acquired, and ventricular spaces were quantified using a series of coronal sections as described above.

### Statistics

Results are expressed as mean ± SEM of the indicated number of experiments. Statistical analysis was performed using Student’s t test, and one- or two-way analysis of variance (ANOVA), as appropriate, with Tukey post hoc tests or Bonferroni post-hoc tests for repeated measurements. A *p*-value equal to or less than 0.05 was considered significant. Number of animals used in each experiment are indicated in Figure legends.

## Results

### Genetic and pharmacological models of microglia ablation led to efficient microglia ablation

**Fig. 1:**
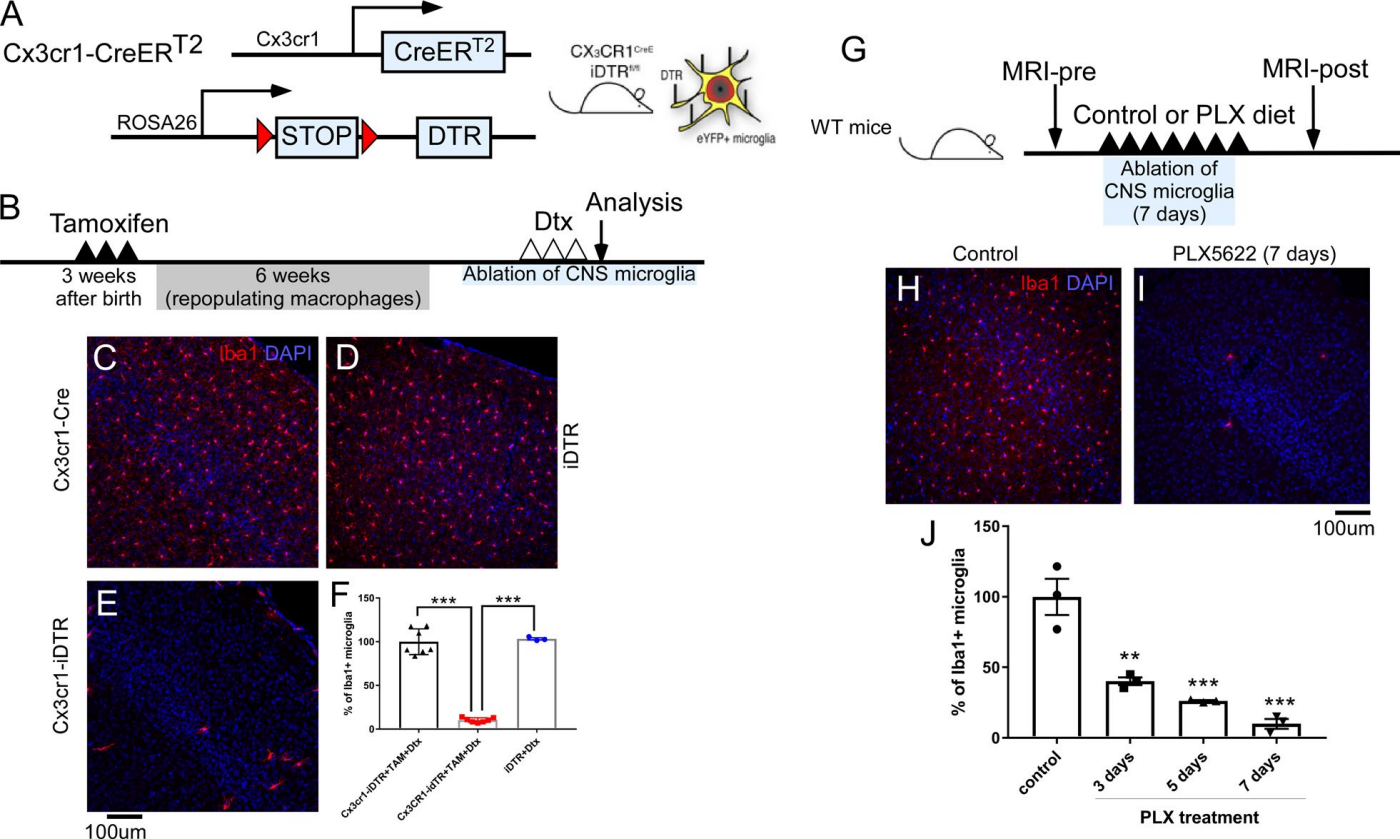
Genetic (Cx3cr1-iDTR) and pharmacological (PLX5622) model of microglia ablation leads to efficient microglia ablation. **A** Generation of genetic microglia ablation model in Cx3cr1-iDTR mice. **B** Experimental timeline for genetic CNS specific microglia ablation. **C** Quantification of microglial cell numbers in control (Cx3cr1-Cre *n* = 7 or iDTR *n* = 3) or microglia ablation mice (Cx3cr1-iDTR *n* = 7, *p* < 0.001, One-way ANOVA). **D** representative images of microglia cell (IBA1-immunostaining) in control and ablated mice. **F** Experimental timeline for PLX5622 mediated microglia ablation. **G**, **H** representative images of microglia cell (IBA1-immunostaining) in control and PLX5622-ablated mice. **I** Quantification of microglial cell numbers in control (control diet for 7 days, *n* = 3) or mice that were subjected to PLX5622 for different days (*n* = 3 for each time points, Mean ± SEM, ** or *** indicates < 0.01 or *p* < 0.001 compared to control, one-way ANOVA). Quantification result is from one cohort of mice with similar results observed from > 2 other independent experiments

Given the recently reported important roles of microglia in multiple aspects of CNS function, we investigated chemical and genetic microglia ablation models, as described in previous studies, to evaluate the role of microglia under physiological and pathological conditions [15, 16]. We first examined the efficiency of microglia ablation in the Cx3cr1-iDTR model. Cx3cr1-iDTR mice were generated by breeding between Cx3cr1CreERT2 mice and ROSA26iDTR mice and treated with tamoxifen 4–6 weeks ahead of Dtx treatment to specifically target microglia, but not peripheral macrophages. Cx3cr1CreERT2 mice that do not have the ROSA26idTR transgene (termed Cx3cr1-Cre mice in this study) were used as control mice. ROSA26iDTR mice that do not have the Cre transgene (termed iDTR in this study) were used and separately analyzed to compared with control mice. As illustrated in Fig. 1A, B, Cx3cr1-Cre mice (*n* = 7), iDTR mice (*n* = 3) and Cx3cr1-iDTR (*n* = 7) mice were subjected to TAM administration to induce the expression of the diphtheria toxin receptor (DTR) in microglia. Diphtheria toxin (Dtx) was then administered 4–6 weeks after TAM-induced DTR expression to specifically target CNS microglia (peripheral macrophages are replenished from Cx3cr1 negative bone marrow progenitors in 6 weeks and, therefore, are no longer DTR +) [15, 16]. This allows for specific CNS microglia ablation, as previously reported [15]. We adopted a widely used treatment dosage/paradigm for Dtx administration (20 ng/g/day × 3 days) and our data demonstrates that after 3 days of Dtx administration, we were able to achieve over 90% microglia ablation (Fig. 1F, *n* = 7) in the Cx3cr1-iDTR mice compared to the two different control mouse groups (Cx3cr1-cre only, *n* = 7 or iDTR only, *n* = 3). Data shown in Fig. 1C–E are representative images from brain cortex and are consistent across different brain regions (data not shown). These results confirm previous findings indicating that the Cx3cr1-iDTR mouse is efficient in ablating microglia within a short time window (3 days) after an initial Dtx treatment. We then assessed the pharmacological ablation model using the specific CSF1R kinase inhibitor, PLX5622. Figure 1G outlines administration of either PLX5622 or a control diet over the course of 7 days to wildtype mice on a C57BL/6 background. Following 7 days of PLX5622, up to 90% microglia were ablated as shown in Fig. 1J. Representative cortex images of IBA1 staining in Fig. 1H, I show efficient microglia ablation in the cortex, which was consistent across brain areas (data not shown). These results support previous reports showing efficient microglia ablation following 7 days of PLX5622 diet [44, 45].

### Genetic microglia ablation results in loss of CSF/ventricular spaces in the brain that is due to the ROSA26iDTR allele but not microglia ablation

**Fig. 2:**
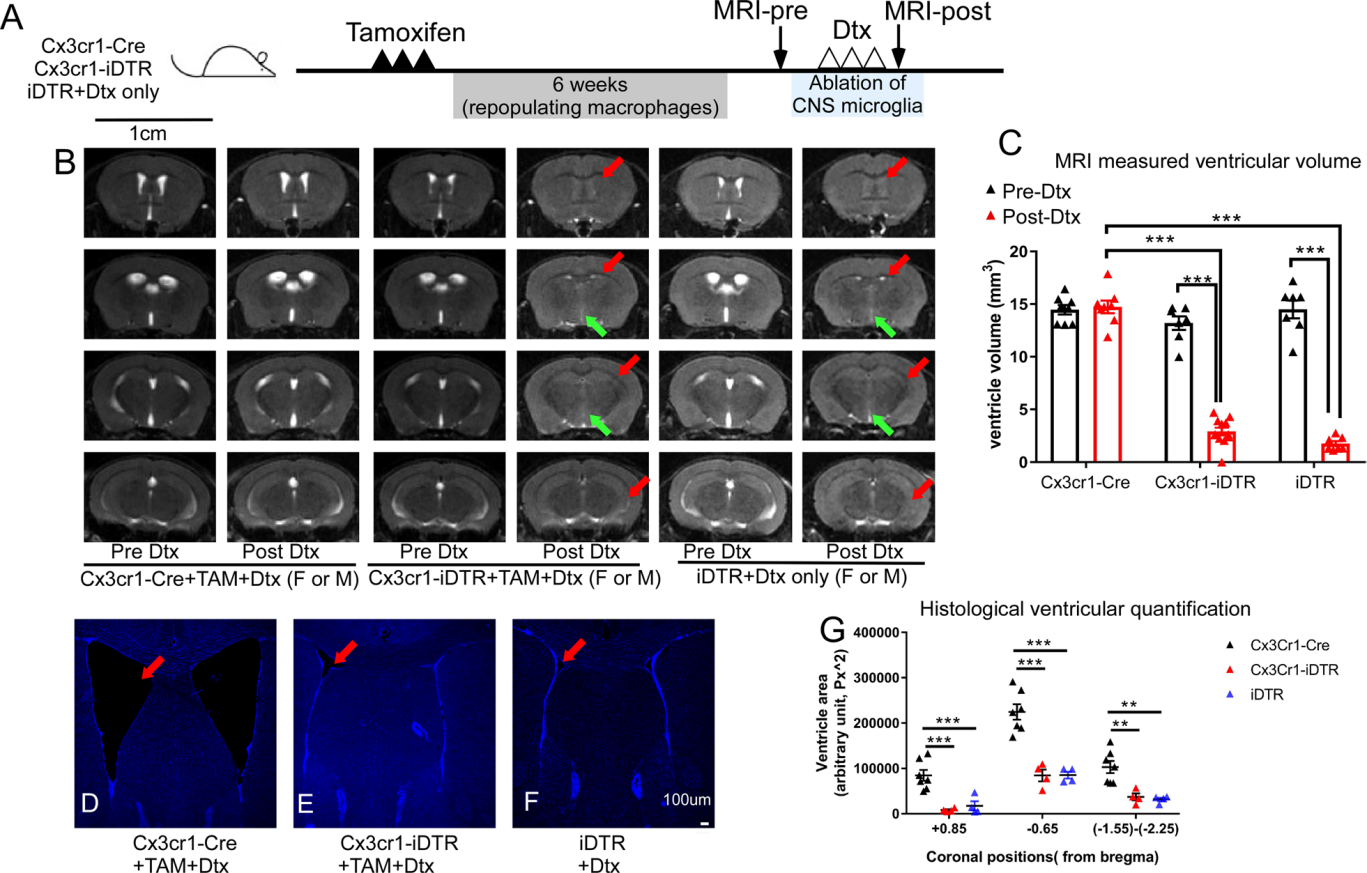
Genetic microglia ablation leads to substantial loss of CSF/ventricular spaces in vivo. **A** Experimental timeline for genetic microglia ablation and MRI quantification. **B** T2-Weighted MRI revealed substantial loss of CSF and lateral ventricular (red arrow) and 3rd ventricular (green arrow) spaces in both female and male microglia ablated mice (Cx3cr1-iDTR, Columns 3 and 4) and the iDTR mice (Columns 5 and 6) after Dtx, whereas Cx3cr1CreER positive but iDTR negative (Cx3cr1-Cre) mice were not affected by TAM and Dtx treatment (Columns 1 and 2). **C** MRI image-based quantification of the ventricular volume in Cx3cr1-Cre, Cx3cr1-iDTR or iDTR mice using serial coronal sections of T2-weighted MRI before and after 3 days of Dtx treatment. **D**, **E**,** F**, Forebrain sections of Cx3cr1-Cre, Cx3cr1-iDTR or iDTR mice subjected to Dtx treatment (red arrow indicating lateral ventricle). Scale bar = 100 μm. **G** histological sections from 3 representative coronal brain sections were used to quantify ventricle areas in the three groups of mice. Consistent with MRI-based quantification, quantification of brain sections shows decreased ventricular/CSF volume in genetic microglia ablated mice (Cx3cr1-iDTR) and the iDTR mice after 3 days Dtx treatment. (Mean ± SEM, ** and *** indicates *p* < 0.01 and 0.001 respectively, Two-way ANOVA with Tukey post-hoc analysis). Quantification result is from one cohort of mice with similar results observed from > 2 other independent experiments

To our surprise, we observed a very robust brain phenotype in the genetic ablation model in T2-weighted MRI. MRI and subsequent ventricular size analysis demonstrated that following Dtx administration, 100% of the microglia-ablated (Cx3cr1-iDTR TAM treated) mice (*n* > 35) showed a substantial loss of CSF and ventricles (both lateral ventricles and the 3rd ventricle, red and green arrows, Fig. 2B) in both female and male Cx3cr1-iDTR mice. Cx3cr1CreER positive but iDTR negative control mice (Cx3cr1-Cre mice) subjected to the same TAM and Dtx treatment were unaffected (*n* > 39). However, ROSA26iDTR only mice (iDTR mice) that do not have any cre transgene showed the same phenotype of CSF and ventricle loss (*n* > 30). Indeed, Cx3cr1-iDTR and iDTR mice who received Dtx without TAM also showed the same phenotype (Fig. 2B, *n* > 4 for each group), confirming that iDTR allele is solely responsible for this phenotype. To ensure this is not due to potential mutation in our in-house breeding colony, we obtained the independently housed iDTR line from JAX and confirmed the same phenotype in there. Representative MRI for both the Cx3cr1-cre only or the iDTR mice (receiving only Dtx) as well as Cx3cr1-iDTR mice (after TAM treatment) before and after Dtx injection are shown in Fig. 2 (female and male Cx3cr1-iDTR or iDTR only mice demonstrated similar results). A cohort of microglia ablated mice (Cx3cr1-iDTR + TAM and Dtx, *n* = 7) and control mice (Cx3cr1-Cre only + TAM and Dtx n = 7, or iDTR only + Dtx *n* = 7) were used to quantify ventricular volume from the T2-weighted MRI. Our results show that TAM and Dtx treatment does not lead to ventricular volume changes in Cx3cr1-Cre control mice that do not carry the iDTR allele; however, TAM and Dtx treatment in Cx3cr1-iDTR mice or Dtx only treatment in iDTR mice led to a substantial decrease of CSF/ventricular volume (Fig. 2C, 70% loss, *p* < 0.001, ANOVA) measured at 1 day after 3 days of Dtx treatment. To confirm the loss of ventricular space in T2-weighted MRI, this phenotype was also validated by histological analysis of Cx3cr1-Cre or iDTR only groups and genetic microglia-ablated (Cx3cr1-iDTR) brain sections (Fig. 2D–F). Histological quantification of the brain section areas demonstrates that at all three representative coronal positions (+ 0.85 mm, − 0.65 mm and − 1.55 to − 2.25 from Bregma), ventricle areas were significantly decreased in the microglia ablated mouse brains or iDTR+Dtx group as compared to Cx3cr1-Cre control littermates (*n* = 4–5 per group and *p* < 0.01 or *p* < 0.001, Fig. 2G). DAPI stained mouse brain sections at the equivalent forebrain position containing the lateral ventricles are presented to clearly show a loss of ventricular space in Cx3cr1-iDTR and the iDTR only groups (Fig. 2D–F). Importantly, the loss of ventricle spaces is not due to either TAM or Dtx administration alone, as Cx3cr1Cre:iDTR^wt/wt^ control mice (Cx3cr1-Cre) that also received TAM and Dtx treatment did not show this phenotype (by MRI or histology). Furthermore, Cx3cr1-iDTR mice expressing DTR on their microglia (that received TAM administration) but were not ablated (i.e., before Dtx injection, or that received vehicle instead of Dtx injection) did not develop this phenotype (Fig. 2B for pre Dtx injection and data not shown for vehicle injection). Since both Cx3cr1-iDTR and iDTR mice receiving just Dtx presented this phenotype, this suggests that the observed loss of ventricular space is specifically due to the iDTR allele and Dtx treatment in this genetic model.

### Pharmacological depletion of microglia does not result in loss of ventricular spaces in the brain

**Fig. 3:**
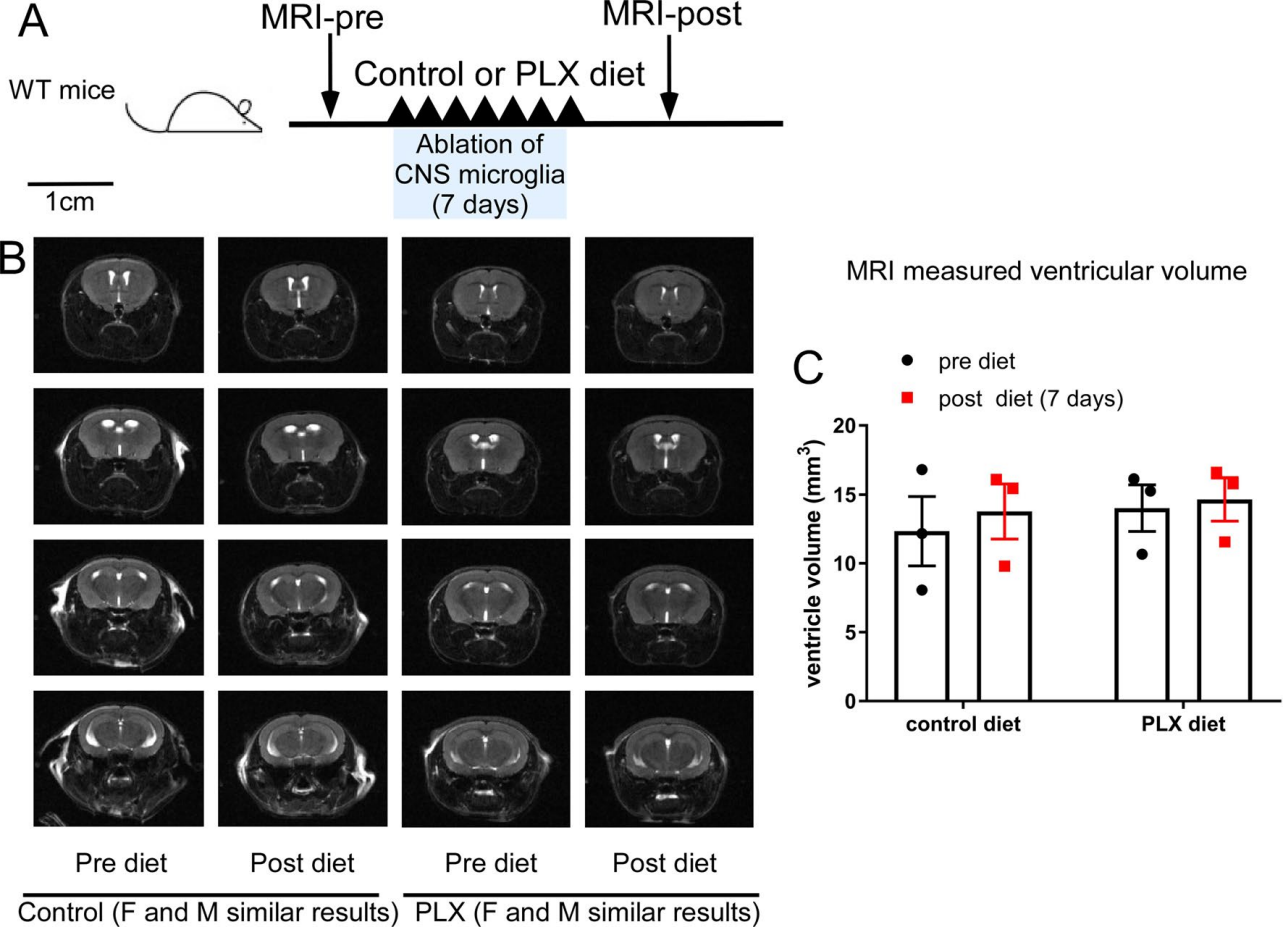
Pharmacological microglia ablation (PLX5622) does not lead to changes in CSF/ventricular spaces in vivo. **A** Experimental timeline for PLX5622 mediated microglia ablation and MRI quantification. **B** T2-Weighted MRI shows no significant difference in control diet or PLX5622 diet mice before or after the diet treatment. **C** MRI image-based quantification of the ventricular volume in control and PLX5622 microglia ablated mice using serial coronal sections of T2-weighted MRI before and after 7 days of control of PLX5622 treatment. (*n* = 3 for each group, Mean ± SEM, and no statistical differences among any of the groups by two-way ANOVA with Tukey post-hoc analysis). Quantification result is from one cohort of mice with similar results observed from > 2 other independent experiments

After observing the robust phenotype in the genetic ablation model, we wanted to investigate whether this pathological condition is due to the loss of microglia, per se, or is specific to the genetic ablation model. Using MRI to compare the ventricular spaces before and after 7 days of PLX5622 or control diet (Fig. 3), CSF/ventricular volumetric changes were not observed after pharmacological microglia ablation (Fig. 3C, n = 3 per group, *p* = 0.536 for diet and *p* = 0.616 for pre vs post diet, Two-way ANOVA). Figure 3B shows representative scans from before and after PLX5622 diet, including data from both males and females, showing similar results. This supports the notion that depleting the microglia population alone is not sufficient to induce the ventricle loss phenotype. Additionally, the mechanism through which PLX5622 depletes the microglia population does not induce the same CSF/ventricle loss phenotype seen in the genetic ablation model.

### Microglia ablation in the Cx3cr1Cre:iDTR model but not the PLX5622 model leads to astrocyte activation and upregulation of multiple cytokines in brain

**Fig. 4:**
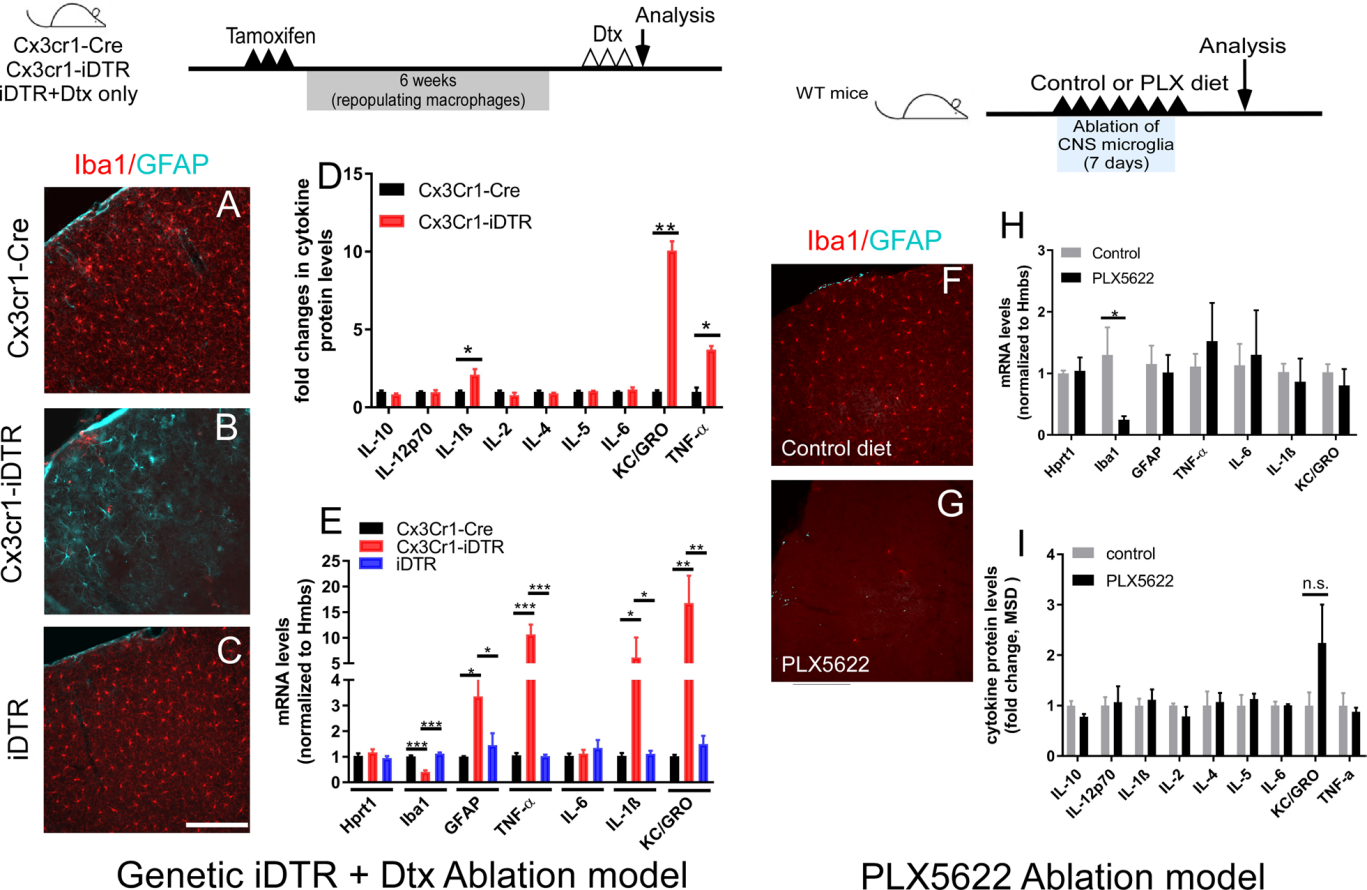
Genetic ablation of microglia in Cx3cr1-iDTR mice leads to activation of astrocytes **A**–**C** and upregulation of multiple cytokines measured by cytokine protein levels (**D**) and mRNA levels (**E**), compared to Cx3cr1-Cre or iDTR mice subjected to Dtx treatment. Scale bar = 100 μm. **F**–**I** PLX5622 microglia ablation does not lead to astrocyte activation. mRNA levels and protein levels of cytokine show no difference between control and microglia ablated mice (PLX5622). (*n* = 6–8 for qRT-PCR and *n* = 3–4 for cytokine measurement, Mean + SEM, *p < 0.05 and **p < 0.01 and ***p < 0.001 one-way ANOVA for E and Student’s *t*-test for **D**, **H** and **I**). Two cohorts of mice were pooled for q-RT-PCR experiments and one cohort of mice was used for cytokine measurement

After confirming that the pharmacological ablation method did not show the same phenotype as the genetic model, we next investigated whether additional cytokines are altered upon acute microglia ablation in the genetic model (either Cx3cr1-iDTR ablation or just iDTR mice with Dtx treatment) vs. pharmacological ablation model. Utilizing immunohistology, we first confirmed successful ablation of microglia and the activation of astrocytes, as established by GFAP upregulation within astrocytes, to be consistent with the prior study [15] in the Cx3cr1-iDTR microglia ablated brains (Fig. 4A, B). qRT-PCR demonstrated that *Iba1* mRNA levels are significantly decreased in Cx3cr1-iDTR microglia ablated mice, consistent with our immunohistology results (Fig. 4E, *n* = 5–7 per group, *p* < 0.001, Student’s *t*-test). In contrast, *Gfap* mRNA levels are significantly upregulated (Fig. 4B and E, *n* = 5–7 per group, *p* < 0.001, Student’s *t*-test), which is consistent with the activation of astrocytes (revealed by an increased GFAP immunoreactivity) in the genetic microglia ablation model. To determine whether cytokines are upregulated in the microglia ablation model, we utilized a multiplex ELISA based approach for targeted discovery using the validated V-Plex inflammatory cytokine panel (Meso Scale Discovery). This panel allows for the evaluation of multiple cytokines in the same sample. Our data showed that of the 10 inflammatory cytokines examined, three of them were significantly increased in microglia ablated (Cx3cr1-iDTR) mouse brains: TNF-α, IL1β and the KC/Gro (Fig. 4D, p < 0.05 or *p* < 0.01, *n* = 4 per group). To investigate whether the increase in these cytokines occurs at a transcriptional level and to additionally assess whether iDTR mice similarly exhibit this increase, we measured mRNA levels in the cortex of all three groups of mice (Cx3cr1-Cre, Cx3cr1-iDTR, and the iDTR mice). Notably, TNF-α, IL-1β and KC/GRO cytokine mRNA levels demonstrated a significant increase in genetic microglia ablated (Cx3cr1-iDTR) mice (Fig. 4E, *p* < 0.01, Student’s *t*-test, *n* = 5–7 per group), consistent with the changes in the cytokine protein levels. Thereafter, we examined whether the iDTR mice that were subjected to Dtx treatment had cytokine upregulation and astrocyte activation, as observed in the Cx3cr1-iDTR ablated brains. Our data show that although we observed the same CSF/ventricle loss in the iDTR mice subjected to Dtx treatment, we do not observe any of the cytokine gene upregulation or astrocytes activation at 1 day after the 3 day Dtx treatment (Fig. 4C, E). We next investigated whether these mRNA and protein level changes in inflammatory cytokines and reactive astrocytes were specific in the microglia genetic ablation model. First, although the microglia ablation efficiency is similar to the genetic ablation model, in the PLX5622 model an increase in GFAP expression (as assessed by immunohistochemistry) was not observed (Fig. 4F–G). Additionally, only a decrease in *Iba1* mRNA levels was evident without changes in the mRNA of *Gfap* or inflammatory cytokines (Fig. 4H). Furthermore, multiplex ELISA assay found no changes in corresponding protein levels (Fig. 4I, *p* > 0.05 for all cytokines, Student’s *t*-test). This suggests that the loss of CSF/ventricular spaces is not due to loss of microglia per se, and that the cytokine upregulation in the Cx3cr1-iDTR microglia ablated brains is not a driving factor accounting for the loss of CSF /ventricle phenotype in the genetic ablation model.

### Loss of ventricular spaces is not a result of increased parenchymal volume or altered brain water content

**Fig. 5:**
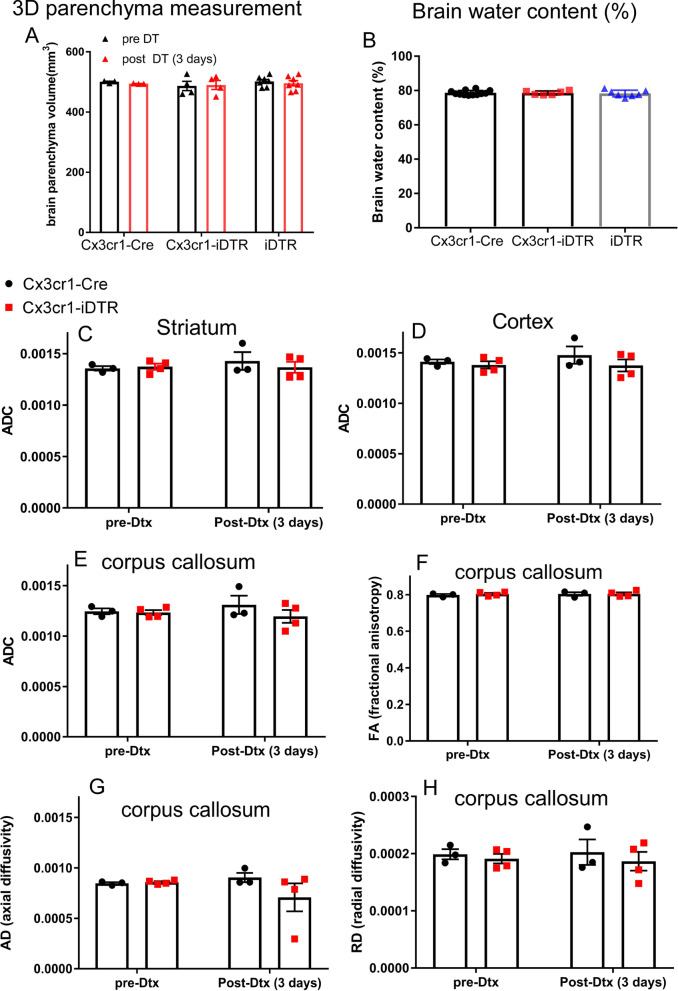
Genetic ablation of microglia in Cx3cr1-iDTR mice does not cause brain parenchyma swelling or edema. **A** MRI measures of 3D parenchyma volume show no difference in genetic microglia ablated mouse brains compared to Cx3cr1-Cre or iDTR brains after Dtx treatment. **B** Water content does not change among Cx3cr1-Cre, Cx3cr1-iDTR or iDTR brains. **C**–**H** Diffusion parameters calculated from DTI data show no differences between microglia ablated (Cx3cr1-iDTR) and control (Cx3cr1-Cre) animals in striatum, cortex and corpus callosum. (Each data point represents an individual animal. Mean + SEM. No significant difference among any of the groups one-way or two-way ANOVA with Tukey post-hoc analysis). **A** and **B** data pooled from two independent experiments and **C**–**H** from a single experiment

Two potential alternative mechanisms may contribute to the observed decrease in CSF/ventricular volume in the iDTR genetic model. Parenchyma swelling could lead to expansion of brain parenchyma to, thereby, reduce ventricular spaces (a phenomenon that can be observed in edema caused by stroke, for example). Alternatively, the loss of ventricular spaces could be caused by decreased CSF production/circulation. After establishing the decreased CSF/ventricle size in the genetic ablation model, we sought to investigate whether parenchymal swelling is present in the microglia ablation model that could potentially contribute to the reduced ventricle size. First, we measured the total parenchyma volume (excluding ventricular volume) of control or microglia ablated mouse brains to investigate whether there is brain tissue swelling and a potential increase in brain parenchyma volume. Overall parenchymal volume was measured using 3D MRI images, and no difference was evident in brain parenchyma volume after administration of Dtx (Fig. 5A, *n* = 3–7, *p* > 0.05, ANOVA) among the three groups (Cx3cr1-Cre or iDTR or Cx3cr1-iDTR mice). As an alternative method to measure potential brain edema/swelling, brain water content was measured in control or microglia genetically ablated mouse brains using methods previously described [37]. As shown in Fig. 5B, there is no difference in total brain water content percentages among the Cx3cr1-Cre, iDTR and the microglia ablation (Cx3cr1-iDTR) groups (Fig. 5B, *n* = 6–9, *p* > 0.05, ANOVA) at 1 day after the last day of Dtx treatment, a time point when ventricular space loss can be readily observed. We additionally used diffusion tensor imaging (DTI) to evaluate water diffusion in the cortex and striatum as well as directionality in the corpus callosum. Similarly, we did not observe differences in diffusion metrics in any location (Fig. 5C–H, *p* > 0.05 for all parameters examined, ANOVA). In summary, different methods of measuring brain tissue swelling or water content and diffusion suggest that the decrease in CSF/ventricular spaces in the iDTR or the Cx3cr1-iDTR microglia ablated mice is not likely due to tissue swelling in these mouse brains.

### The KC/GRO pathway is involved in brain edema formation in an experimental stroke model but does not resolve shrinking ventricles after genetic ablation of microglia

**Fig. 6:**
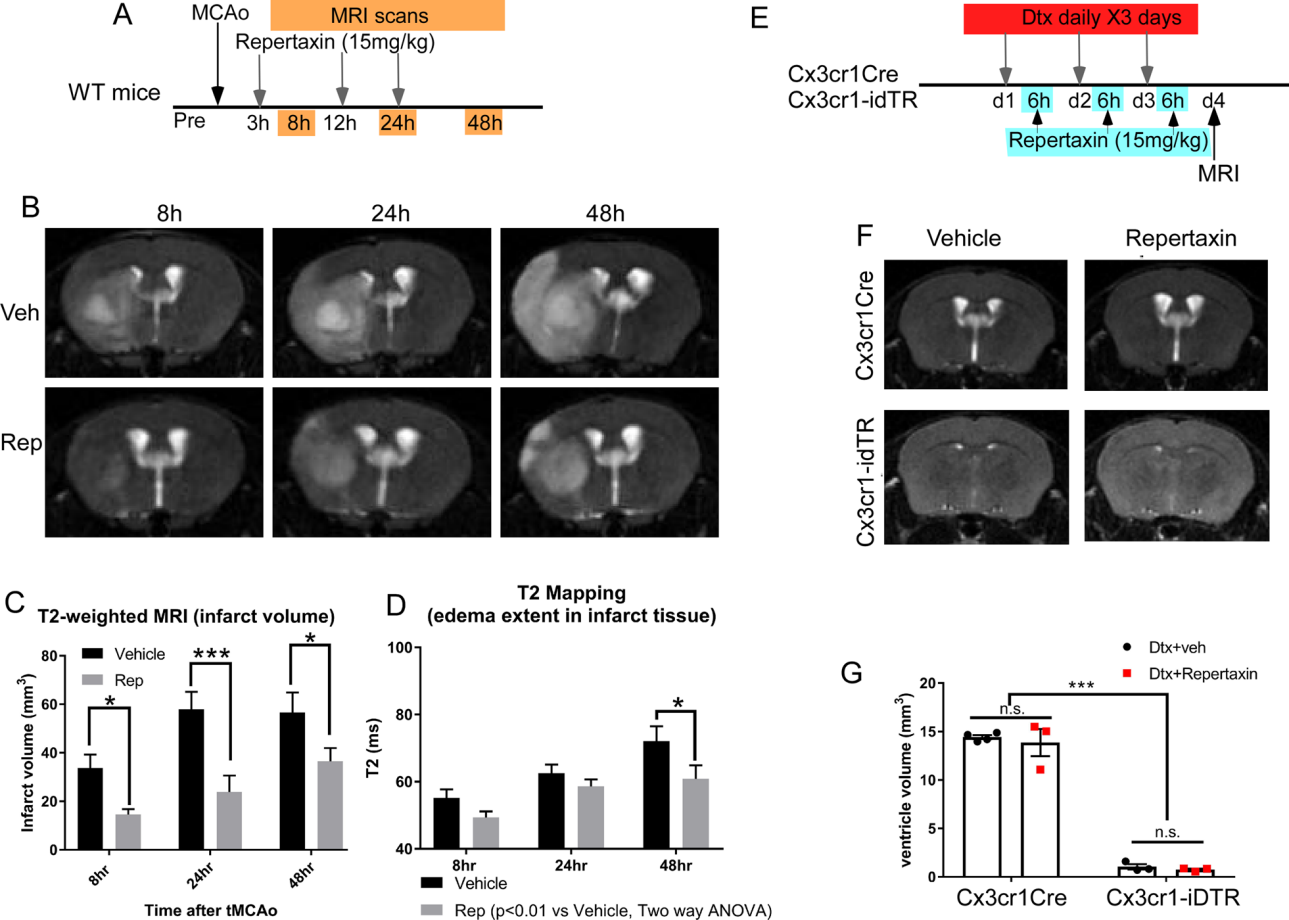
Inhibition of the KC/Gro pathway reduces stroke infarct volume and edema but does not reverse the CSF/ventricle shrinkage phenotype in the genetic microglia ablation model. **A** experimental timeline for stroke experiment. **B** Representative T2-weighted MRI imaging of vehicle or Repertaxin (Rep) treated mice at 8, 24 and 48 h after stroke. **C** infarct volume measured by T2-weighted MRI images showed decreased infarct volume in Repertaxin treated mice (*n* = 9) compared to Vehicle treated mice (*n* = 6). **D** Repertaxin treatment also decreased the T2 relaxation time, which is consistent with a reduction in water content. (Two- way ANOVA, treatment *p* < 0.001 for infarct volume and *p* < 0.01 for T2 mapping. * or ** and *** indicates *p* < 0.05, 0.01 and 0.001 for Bonferroni post-hoc tests for repeated measurements). **E** Experimental timeline for genetic microglia ablation and Repertaxin treatment. **F** Representative T2-weighted MRI images showing shrinkage of CSF/ventricular spaces in vehicle and Repertaxin treated microglia ablated mouse brain. **G** MRI image-based quantification of the ventricular volume in control and genetic microglia ablated mice treated with vehicle or Repertaxin using serial coronal sections of T2-weighted MRI after 3 days of Dtx treatment. (*n* = 3–4 for each group, Mean + SEM, ***p < 0.001, two-way ANOVA with Tukey post-hoc analysis). Data were pooled from two independent experiments showing similar results for both experimental paradigms

While analyzing brain swelling and water content/diffusion in the genetic microglia ablation model, we also explored the potential contribution of the less studied KC/Gro pathway in the observed CSF/ventricular shrinkage phenotype. Of the three cytokines that are elevated in the genetic microglia ablation model, TNF-α and IL1β have been shown to mediate brain inflammation and edema by us and others [46–50]. We therefore decided to focus on the third cytokine, KC/Gro, whose role is less well understood but is starting to gain interest in neurological diseases. The chemokine receptor CXCR2 and its ligands have been implicated in a variety of peripheral inflammatory diseases [51] and, more recently, have been linked to CNS disorders [52–54]. We examined the efficacy of blocking KC/GRO, the most upregulated candidate cytokine revealed by both RT-PCR and ELISA following genetic ablation of microglia (Fig. 4), to mitigate brain CSF/ventricular shrinkage. Specifically, an inhibitor (Repertaxin) to the receptor of the KC/Gro cytokine, CXCR2, was used. Repertaxin is a non-competitive allosteric inhibitor for CXCR2 and has been shown to be neuroprotective in rodent stroke models in two previous studies [43, 55]. We utilized a previously established Repertaxin dosage [41] to inhibit the CXCR2 signaling pathway during each day of Dtx administration (6 h after administration to avoid drug interactions) to assess whether blockade of KC/GRO signaling was sufficient to resolve the ventricle shrinking phenotype observed in the genetic microglia ablation model. We validated the efficacy of the Repertaxin dosage on a stroke model which showed decreased edema of the stroke mice treated with Repertaxin (Fig. 6A–D). As shown in Fig. 6, Repertaxin treatment in stroke mice decreased brain edema but did not prevent or improve the phenotype previously seen in the genetic ablation model (Fig. 6F–G, *n* = 3–4, *p* < 0.001 for control vs. genetic microglia ablation; *p* > 0.05 for vehicle vs. Repertaxin, ANOVA). This data supports the notion that the loss of ventricular space phenotype in the genetic microglia ablation model is not likely due to edema and cannot be reversed by blocking the KC/GRO pathway.

### Loss of ventricular spaces in Cx3cr1-iDTR mice or iDTR mice does not lead to neurodegeneration in cortical layers at 10 days after the last Dtx injection

**Fig. 7:**
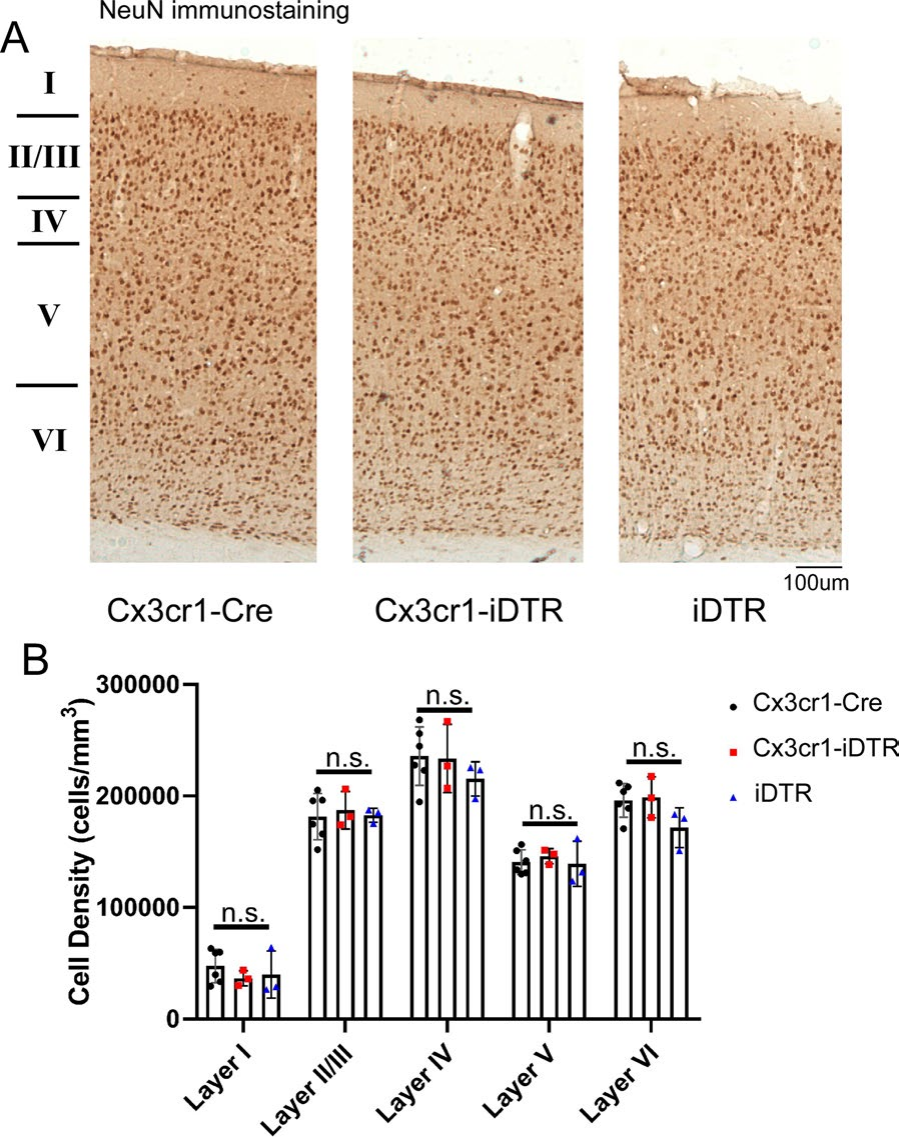
Genetic ablation of microglia in Cx3cr1-iDTR mice does not lead to neurodegeneration in the somatosensory and motor cortex. **A** Representative cortical images showing NeuN staining at 1 day after a 3 day Dtx treatment regimen. **B** Quantification of NeuN stereological estimates for cell density in each cortical layer. No statistical difference was found among groups at any cortical layer (*N* = 3–6 for each group, Mean ± SEM, One-way ANOVA for each layer among three groups). Quantification result is from one cohort of mice with similar results observed from > 2 other independent experiments

One previous study has reported that acute microglia ablation using the Cx3cr1-iDTR mouse model leads to neurodegeneration of cortical layers at 10 days after the last Dtx injection [24]. To examine whether we observe similar neurodegeneration in our Cx3cr1-iDTR mouse model or in our iDTR mice that do not have microglia ablation but show ventricular space/CSF loss, we carried out unbiased stereology counts of all layers of somatosensory cortex in Cx3cr1-Cre, Cx3cr1-iDTR and iDTR mice subjected to Tamoxifen and Dtx treatment. At the same time point as previously reported (10 days after the last Dtx injection), we did not observe any differences in NeuN + neuronal density in the cortical layers (Fig. 7), suggesting that neither microglia ablation nor the loss of CSF/ventricular space at this time point leads to neurodegeneration.

### Activated IBA1 positive cells are observed surrounding the ventricular spaces and within choroid plexus following genetic ablation of microglia

**Fig. 8:**
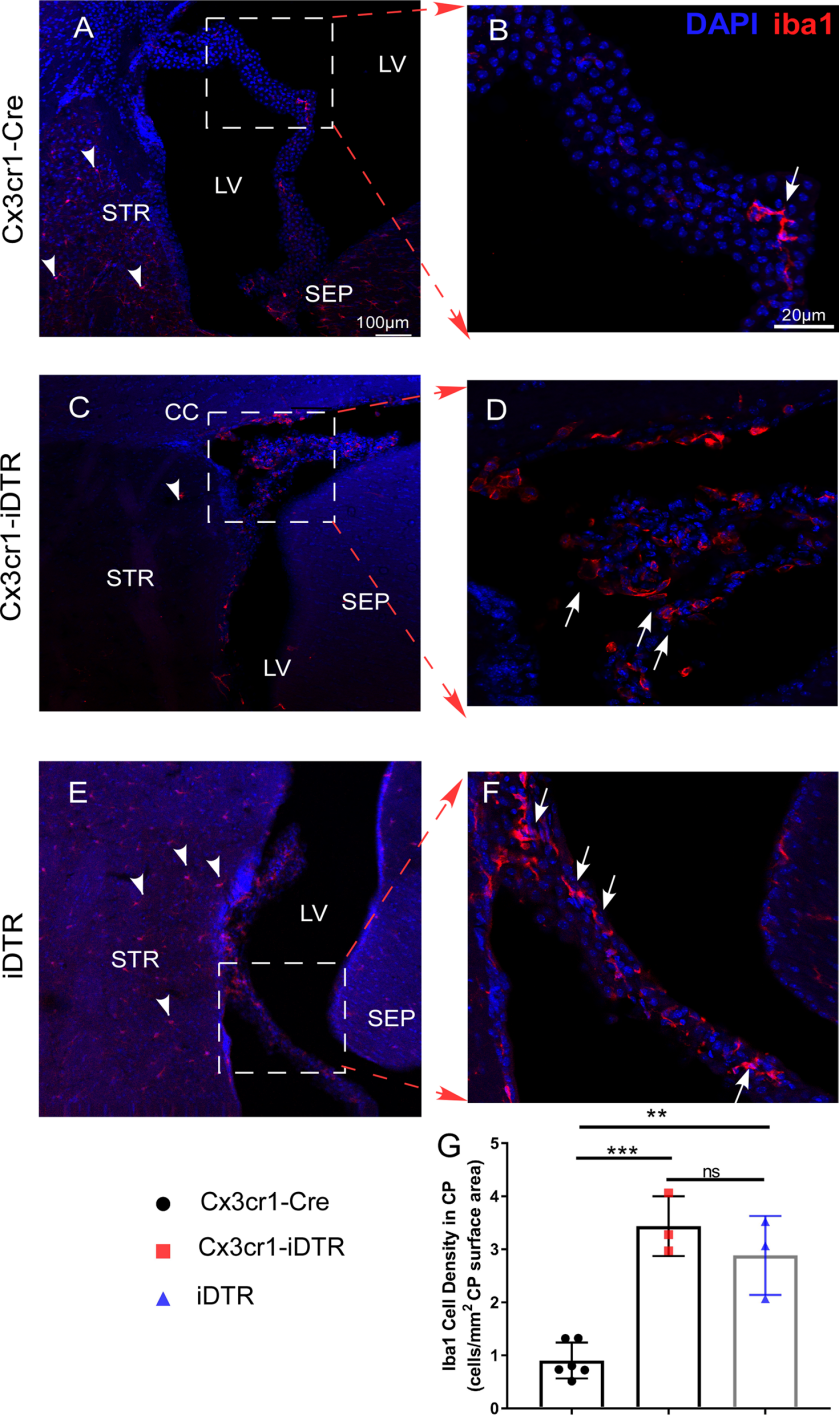
Increased IBA1 + cells are present in choroid plexus (CP) in Cx3cr1-iDTR and the iDTR mice after 3 day Dtx treatment. Genetic ablation of microglia in Cx3cr1-iDTR mice leads to ablation of IBA1 + microglia in the brain parenchyma (indicated in **A** and **C** by arrowheads). However, in the choroid plexus (CP) it leads to increased IBA1 + cells (indicated by arrows in **B** and **D**), suggesting a potential inflammatory response and possible infiltration of peripheral macrophages into CP. iDTR mice subjected to Dtx treatment does not result in microglia ablation (indicated in **E** by arrowheads) but likewise results in increased IBA1 + cells in CP, similar to Cx3cr1-iDTR mice (scale bar = 100 μm left column, scale bar = 20 μm right column). (*n* = 3–6 for each group, Mean ± SEM, **p < 0.01 and ***p < 0.001, one-way ANOVA). Quantification result is from one cohort of mice with similar results observed from > 2 other independent experiments

After establishing that brain swelling/edema is not likely a cause for the observed the ventricular space loss in the genetic microglia ablation model, we investigated other potential avenues that could explain the resulting phenotype. The fact that iDTR mice receiving Dtx also develop this loss of CSF/ventricle phenotype without upregulation of cytokines and reactive astrocytes further supports the notion that production/circulation of CSF might be contributing to this pathology rather than edema or brain swelling. In this light, the next region of interest was the choroid plexus (CP) as it is recognized to be a major source for CSF production in the brain [56]. We examined the IBA1 + cells in CP during the period when ventricular spaces were substantially reduced in both iDTR and Cx3cr1-iDTR mice (d4 after the first Dtx injection). Immunostaining of IBA1 in CP revealed an increased number of IBA1 + cells in both the iDTR and Cx3cr1-iDTR microglia ablated animals. In the microglia ablated mice (Cx3cr1-iDTR), despite a decrease in IBA1 positive cells, validating successful ablation of microglia, in the parenchyma (Fig. 8C, positive cells in parenchyma highlighted with arrow heads), the CP and ventricular wall showed an enriched IBA1 positive population following microglia ablation (representative images in Fig. 8D, positive cells in the CP highlighted with arrows quantification in Fig. 8G, *p* < 0.001, ANOVA). In iDTR mice subjected to Dtx treatment, we did not observe any ablation of IBA1 + microglia in the parenchyma (Fig. 8E, positive cells highlighted with arrow head) but observed a similar increase in the IBA1 positive population in the CP following Dtx treatment (representative images in Fig. 8F, positive cells in the CP highlighted with arrows quantification in Fig. 8G, *p* < 0.01, ANOVA), Additionally, the IBA1 positive cells in the CP in microglia ablated brains appear less ramified than homeostatic microglia, which is suggestive of an activated microglia or macrophage origin. This data suggests that, instead of brain tissue swelling, potential pathology in the CP which is reflected by increased activated IBA1 + cells might contribute to the observed ventricular shrinkage phenotype observed in the iDTR and the Cx3cr1-iDTR microglia ablation model. The precise mechanism(s) of pathology in the CP and the effects on various CP cellular components is currently under further investigation.

## Discussion

With the goal of understanding the role of microglia under physiological and various pathological conditions, two novel microglia ablation models have recently been reported, either genetically targeting microglia [15–17] or through pharmacologically targeting the CSF1 receptor (CSF1R) with PLX3397 or 5622 [13, 14, 18–20]. Although both models effectively ablate microglia and have been demonstrated to be valuable tools in the field, a major difference between the two systems is that whereas the genetic ablation model can lead to the upregulation of select cytokines and induces astrocyte activation [15], the pharmacological model, in general, appears not to cause such actions [18, 57]. The upregulation of cytokines and astrocyte activation in the genetic ablation model may cause additional deficits in the brain that are not specifically related to microglia function. In this study, we describe a consistent loss of ventricle pathology in both the Cx3cr1-iDTR and iDTR mouse models following Dtx administration that has not been reported previously.

Given the wide utilization of the microglial ablation models and the subsequent large number of high impact publications using these models [13–20, 22–25], it is important to carefully characterize these models utilizing multiple analyses and methods. The genetic model of microglia depletion has been used in over 700 publications since its conception. It has been used to investigate microglia repopulation in age-dependent studies to better understand mechanisms behind adult microglia replenishing [15]. This model has also been used to investigate how microglia play a role in learning and memory [16]. Additionally, it has also been used in a variety of neurological disorder-focused studies, including Alzheimer’s disease, seizures, and stroke [58–60]. The PLX model of microglia ablation has similarly been used to examine stroke, Alzheimer’s disease, and Parkinson’s disease [13, 14, 21, 61]. In our characterization of the genetic microglia ablation model, we discovered that Cx3cr1-iDTR mice develop a robust, unpredicted neuropathology, distinguished by a significant loss of ventricular spaces (Fig. 2), which was not observed in the pharmacological ablation model (Fig. 3); thereby suggesting that this pathology is not due to ablation of microglia per se, but, rather, represents a specific pathology observed in the genetic ablation model. We further discovered that this pathology is intrinsic to the ROSA26iDTR (iDTR) allele following Dtx treatment independent of cre expression. This pathology is not associated with the observed cytokine upregulation or the reactive astrocytes as the iDTR group subjected to Dtx that, likewise, exhibits a loss of CSF/ventricle phenotype, does not show increased cytokine or reactive astrocytes. Consistent with this observation, through 3D MRI analysis, histological analysis, and brain water content analysis, our data suggests that the loss of ventricles is not a result of parenchymal swelling. Additionally, inhibition of the KC/GRO signaling pathway, which demonstrated success in reducing edema severity and infarct development in a severe MCAo stroke model (Fig. 6A–D), was ineffective in preventing the induction of ventricle loss in the genetic microglia ablation model. Alternatively, our data suggests a CP-related pathology that is revealed by enrichment of activated IBA1 + positive cells in the CP in both iDTR mice and Cx3cr1-iDTR mice subjected to Dtx treatment. Currently, we are undertaking further detailed studies to characterize the precise cellular and molecular mechanisms contributing to this CP related pathology. Until the precise cause of pathology is established, caution is warranted when utilizing the genetic ablation model to study subtle neurological functions using Dtx mediated genetic ablation models and, particularly, the CNS cell-type specific ablation model. Specifically, caution needs to be given when utilizing this model to interpret subtle neurological functional changes that are thought to be mediated by microglia/or other neural cell types as these could, instead, be due, in part or wholly, to the CSF/ventricular loss pathology [16, 24]; this is of particular importance in light of recent studies indicating the potentially important roles of CSF circulation in homeostasis and pathological conditions of the brain [62–66]. Since ROSA26iDTR allele is responsible for the pathology after Dtx administration independent of cre expression, this phenotype is likely present in all mouse genetic cell ablation studies utilizing this mouse strain attempted to target specific-cell types with different cre drivers. Despite the wide application of this genetic ablation model, the whole brain MRI was not utilized in prior studies, likely explaining the lack of any earlier report on this pathology/phenotype. Nevertheless, the prospective confounding effect of this genetic ablation model is starting to gain attention, as one recent paper [24] by Rubino et al. reported neuronal cell death (at 10 days post-ablation) caused by genetic ablation of microglia. Interestingly, our data in the Cx3cr1-iDTR microglia ablated brain or iDTR + Dtx brain does not show neuronal cell loss across different cortical layers (Fig. 7), which is consistent with another previous study [16]. The reason for this discrepancy, in relation to our results plus those of Parkhurst et al. (reporting no neurodegeneration in the Cx3cr1-iDTR model) versus the Rubino et al. study remains unclear but could be due to a different dosage of Dtx (we used a lower dosage of 20 ng/g of Dtx versus the 1ug Dtx/mouse used in the Rubino et al. study) or method of neurodegeneration quantification (we used unbiased stereological counts of NeuN + cell density and the Parkhurst et al. study also evaluated NeuN + cell counts versus the immunoreactivity area fraction quantification used in Rubino et al. study). To the best of our knowledge, the work by Rubino et al. [24] is the only published study reporting neuronal cell death and behavioral deficits related to this model. Further validation by additional independent groups is hence needed to examine neuronal cell death immediately after microglia ablation in this genetic model. Nevertheless and notable, the effects on ventricle volume decrease have not been reported previously, thereby making our observation novel and providing a new avenue for research and independent validation. We speculate that the CP related pathology and dysfunction of CSF production could potentially contribute to the observed pathology in the ROSA26iDTR and Cx3cr1-iDTR mice.

## Conclusions

In summary, our data suggests that the genetic microglia ablation model causes an upregulation of multiple cytokines and loss of ventricular spaces accompanied by microglia and astrocyte reactivity that warrant caution when using this model to analyze complex neuronal functions. Our data showed that the pharmacological ablation model (PLX5622) does not cause this particular pathology. Additionally, we discovered that this pathology is also present in ROSA26iDTR mice following Dtx treatment even in the absence of cre expression and independent of cytokine upregulation or reactive astrocytes. We further conclude that this pathology is not a result of parenchymal swelling and, instead, may be related to CP pathology in Dtx treated mice carrying the ROSA26iDTR allele.

## Data Availability

The datasets used and/or analyzed during the current study are available from the corresponding author on reasonable request.

## Abbreviations

AD: Axial diffusivity
ADC: Apparent diffusion coefficient
ANOVA: Analysis of variance
BCA assay: Bicinchoninic acid assay
CNS: Central nervous system
CSF: Cerebrospinal fluid
CSF1R: Colony stimulating factor 1 receptor
CXCL1: The chemokine (C-X-C motif) ligand 1
CXCR2: CXC chemokine receptor 2
Dtx: Diphtheria toxin
DTI: Diffusion tensor imaging
ECA: External carotid artery
ELISA: Enzyme-linked immunosorbent assay
FA: Fractional anisotropy
FAM: 6-Carboxyfluorescein
FOV: Field of view
GFAP: Glial fibrillary acidic protein
HMBS: Hydroxymethylbilane Synthase
HPRT1: Hypoxanthine phosphoribosyl transferase 1
IBA1: Ionized calcium binding adaptor molecule 1
ICA: Internal carotid artery
iDTR: Inducible diphtheria toxin receptor
IFN-γ: Interferon gamma
IL-1β: Interleukin-1β
IL-2: Interleukin 2
IL-4: Interleukin 4
IL-5: Interleukin 5
IL6: Interleukin 6
IL-10: Interleukin 10
IL-12: Interleukin 12
KC/GRO: Keratinocyte chemoattractant /human growth-regulated oncogene
MCAo: Middle cerebral artery occlusion
MRI: Magnetic resonance imaging
MS: Multiple sclerosis
PFA: Paraformaldehyde
RD: Radial diffusivity
RT-PCR: Reverse transcription polymerase chain reaction
TAM: Tamoxifen
TBI: Traumatic brain injury
TE: Echo time
TNF-α: Tumor necrosis factor alpha
TNFR1: Tumor necrosis factor receptor 1
TNFR2: Tumor necrosis factor receptor 2
TR: Repetition time
